# Study of Intra-Chamber Processes in Solid Rocket Motors by Fiber Optic Sensors

**DOI:** 10.3390/s21237836

**Published:** 2021-11-25

**Authors:** Andrey A. Zhirnov, Konstantin V. Stepanov, Stanislav G. Sazonkin, Tatyana V. Choban, Kirill I. Koshelev, Anton O. Chernutsky, Alexey B. Pnev, Alexey O. Novikov, Dmitriy A. Yagodnikov

**Affiliations:** 1 Bauman Moscow State Technical University, 2-nd Baumanskaya 5-1, 105005 Moscow, Russia; a.zh@bmstu.ru (A.A.Z.); sazstas@bmstu.ru (S.G.S.); chobantv@yandex.ru (T.V.C.); koshelev-k@yandex.ru (K.I.K.); chernutsky.a@bmstu.ru (A.O.C.); pniov@bmstu.ru (A.B.P.); novikovao@bmstu.ru (A.O.N.); daj@bmstu.ru (D.A.Y.); 2 Kotelnikov Institute of Radioengineering and Electronics of RAS, Mokhovaya 11-7, 125009 Moscow, Russia

**Keywords:** Mach–Zehnder interferometer, fiber optic sensor, solid fuel, solid rocket motors

## Abstract

In this study, an experimental study of the burning rate of solid fuel in a model solid propellant rocket motor (SRM) E-5-0 was conducted using a non-invasive control method with fiber-optic sensors (FOSs). Three sensors based on the Mach–Zehnder interferometer (MZI), fixed on the SRM E-5-0, recorded the vibration signal during the entire cycle of solid fuel burning. The results showed that, when using MZI sensors, the non-invasive control of solid fuel burnout is made possible both by recording the time of arrival of the combustion front to the sensor and by analyzing the peaks on the spectrogram of the recorded FOS signal. The main mode of acoustic vibrations of the chamber of the model SRM is longitudinal, and it changes with time, depending on the chamber length. Longitudinal modes of the combustion chamber were detected by MZI only after the combustion front passed its fixing point, and the microphone was unable to register them at all. The results showed that the combustion rate was practically constant after the first second, which was confirmed by the graph of the pressure versus time at the nozzle exit.

## 1. Introduction

In modern engines and, particularly, in a solid propellant rocket motor (SRM), energy conversion processes are characterized by extreme temperatures and released power. For example, the thermodynamic temperature is around 3600 K in the combustion chamber of the Ariane-V launch vehicle’s accelerator, EAP P241, which produces a thrust of 7.08 MN [1]. Under such conditions, the SRM operation parameters change frequently due to rates of the main intra-chamber processes—for example, fuel combustion. Therefore, we can describe the working process parameters in SRM as fast-flowing. They include vibrations, pressure in the combustion chamber, and acousto-optical and electrophysical characteristics [2,3].

There are various ways to monitor these parameters. Some methods include rather complex installations using, for example, X-ray analysis [4,5,6] for intra-chamber process control. This method allows for the observation of the fuel mass combustion patterns through the walls of the housing [7]. However, the sampling frequency is low, and the complexity of the setup makes this method inaccessible for common usage. There are also methods for the invasive monitoring of such processes, for example, by inserting thermocouples into test holes. This is simpler, but violates the integrity of the engine and probably changes the operation’s parameters. Additionally, the connection wires require a lot of space, add mass to the setup, and can produce sparks. If we place thermocouples on the motor casing in a non-invasive way, the measurement obtained will be incorrect due to low thermal conductivity in the casing in the outer direction, sensor inertia, and the ambient temperature. In such conditions, fiber-optic sensors (FOSs) present a very promising solution for verifying the simulation data of engine processes [8]. Nowadays, many tasks can be solved in science and technics with the help of FOSs. They are useful for measuring deformation [9], temperature [10,11,12], vibration [13], concentration of substances [14], rotation speed [15], refractive index [16], pressure [17], liquid level [18], acceleration [19], acoustics [20,21,22], and other parameters [2,23,24,25]. Additionally, new types of fiber promise to open new opportunities for such monitoring devices [26,27]. The main FOS related advantage for SRM monitoring is its insensitivity to any kind of electromagnetic interference. Optical fibers, unlike wired connection sensors, cannot originate ignition or explosion, since sparking is excluded during the operation. Optical fibers are produced from dielectric materials, which allow measurements of objects with high electrical voltage to be obtained, and allow for their use in liquid media and environments with high humidity. The chemical inertness of FOS materials allows them to be used under the influence of aggressive, gaseous, and dusty environments, which are realized during tests of SRM. In addition, FOSs that are made of special radiation-resistant fibers can be used when background radiation is high [28]. These performance advantages and their constant evolution predict the rapid deployment of fiber sensors in various aerospace applications [29].

Nowadays, FOSs can be directly integrated into the composite materials of aircraft construction [30]. Fiber Bragg gratings (FBG) are the main sensors used in motors. They detect delamination in the composite housings of rocket engines [31,32]. Polymer fibers [33] and fixed-in-fuel FBGs [34,35,36] have been used to control the state of solid fuel, but this research was conducted during storage and cannot provide information about the working process. Fiber sensors have been used to study rapidly occurring combustion, deflagration, and detonation in SRM [37,38]. Additionally, a spectroscopic analysis of the combustion process was performed using FOSs [39]. Despite the wide range of FOS applications, areas of SRM ignition and combustion processes have not been investigated as of yet. In comparison with X-ray, a simple installation for non-invasive control can be created, with the help of FOSs to control the working process.

In this paper, we propose the concept and operating principle of an FOS based on an Mach–Zehnder interferometer (MZI) for monitoring the combustion of solid fuel. It allows for the registration of combustion front displacement via two principles. The first is based on the housing deformation measurement, and the second uses the registration of acoustic longitudinal modes to measure the combustion chamber parameters. These investigations use the same MZI interference signal, but with different modes of processing.

## 2. Theory

Pressure, temperature, deformation, and vibration constitute the most important parameters of the working process in a model SRM chamber. Currently, only a limited number of FOSs are able detect these values [28,40,41]. The available data suggests the following requirement: an expected total measurement time of less than 10 seconds; typical oscillation frequencies of intra-chamber processes starting from tens of Hz, increasing to hundreds of kHz. In this case, optical time-domain reflectometry (OTDR) systems and devices based on FBG are insufficient due to the low sampling frequency of 30 kHz for phi-OTDR [42,43,44] and 10 kHz for FBG in configurations where there are a few sensors in one line [45,46,47]. In turn, the small SRM length and the requirement of less sensing points allows for the application of sensors based on the MZI. A phase-unwrapping technique was used for dynamic range improvement, based on the 3 × 3 output splitter providing phase-shifted signals.

**Figure 1:**
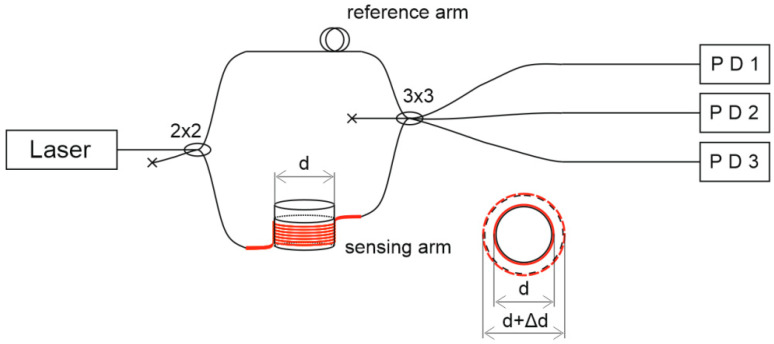
Scheme for the deformation measurement using an MZI.

A fiber MZI registers a phase difference between two arms, one of which is a reference and the other a sensing arm, as shown in Figure 1. No load is applied to the reference arm during the experiment. The sensing arm is fixed on the controlled object, and its length changes depending on the object’s deformation. This leads to a signal intensity fluctuation, which is proportional to the cosine function of deformation. A 3 × 3 output splitter produces a 2*π*/3 phase shift between neighboring fibers. Thus, the recorded intensity on each photoreceiver can be determined by the following expression [48]:

(1) [IPD1(t)=I1+I2+2I1·I2·cos2(πλΔ(t)+φ0),    IPD2(t)=I1+I2+2I1·I2·cos2(πλΔ(t)+φ0+2π3),IPD3(t)=I1+I2+2I1·I2·cos2(πλΔ(t)+φ0−2π3),

where *I*_1_, *I*_2_ are the intensities from the reference and sensing arms, respectively; λ is the wavelength of laser radiation, m; Δ(*t*) is the optical path difference between the MZI arms, m and *φ*_0_ is the initial phase difference, rad.

The presence of two or more signals with a 2*π*/3 phase shift allows for the restoration of the phase *φ* of the deformation without uncertainty caused by a cosine function. We implemented a scheme with two photoreceivers for each MZI, reduced the number of photoreceivers, and simplified the measurement scheme. The deformation phase change Δ*ϕ* was obtained by the algorithm described in [49]. In this case, at each time *t*, it was calculated by the formula:

(2) $$\left(t\right)=\int_{0}^{t}\left[S_{1}\left(t\right)\cdot S_{2}^{’}\left(t\right)-S_{2}\left(t\right)\cdot S_{1}’\left(t\right)\right]dt,$$

where $S_{1}\left(t\right)=I_{PD1}\left(t\right)-I_{PD2}\left(t\right)$, $S_{2}\left(t\right)=I_{PD1}\left(t\right)+I_{PD2}\left(t\right)$.

The phase change depends on the deformation of the fiber, which was influenced by thermal, mechanical, and acoustic effects. In the experiment, the fiber was coiled in certain places on the outer surface of a model SRM, as shown in Figures 1 and 3. The total sensitive fiber length was *L*_sens_ = *Nπd*, where *d* = 19 mm is the initial outer diameter of the SRM housing and *N* = 10 is the number of turns. The SRM diameter increase, and phase change are linked through the fluctuations of the sensing arm length Δ*L*_sens_ by the formula:

$$\Delta \varphi =\frac{2\pi n\Delta L_{\mathrm{sens}}}{\lambda }=\frac{2\pi n\left(N\pi \Delta d\right)}{\lambda }=\frac{2\pi^{2}nN\Delta d}{\lambda },$$

where *n* is the effective refractive index of the fiber core, and consequently,

(3) $$\Delta d=\frac{\Delta \varphi \lambda }{2n\pi^{2}N}.$$

**Figure 2:**
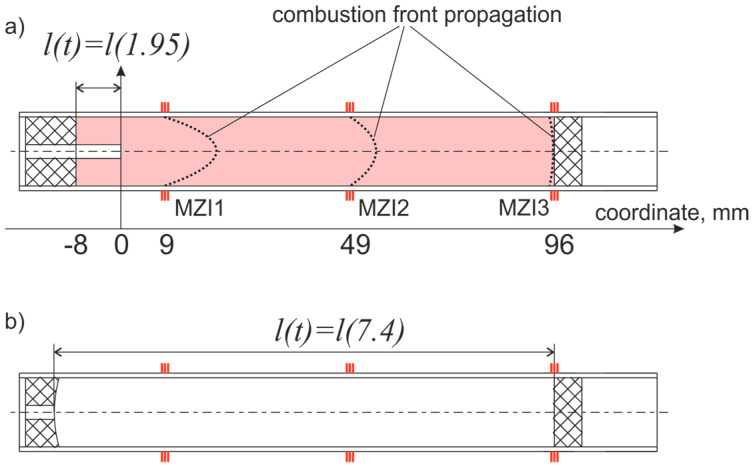
Combustion chamber scheme before (**a**) and after (**b**) work.

A frequency analysis of the phase change provides additional information about the combustion process. The SRM chamber’s acoustic vibration modes depend on its size. The main types are the first longitudinal *f_lon_*, tangential *f_tan_,* and radial *f_rad_*, determined by the formula [50,51,52]:

(4) $$[\begin{array}{c} \begin{array}{c} f_{lon}=\frac{a}{2l\left(t\right)}\\ f_{tan}=0.586\frac{a}{d_{i}} \end{array}\\ f_{rad}=1.22\frac{a}{d_{i}} \end{array},$$

where *a* is the speed of sound, m/s, and *l(t)* is the combustion chamber length, m, at time *t*, s, as shown in Figure 2. Length *l*(*t*) varied from 8 to 109 mm during the experiments; *d_i_* = 15 mm is the chamber internal diameter.

These frequencies contribute to phase change fluctuations and can be observed in the spectra. This observation method can precisely determine the chamber length.

## 3. Experiment

### 3.1. Description of the Experimental Setup and Methods for Recording the Characteristics of Intra-Chamber Processes

**Figure 3:**
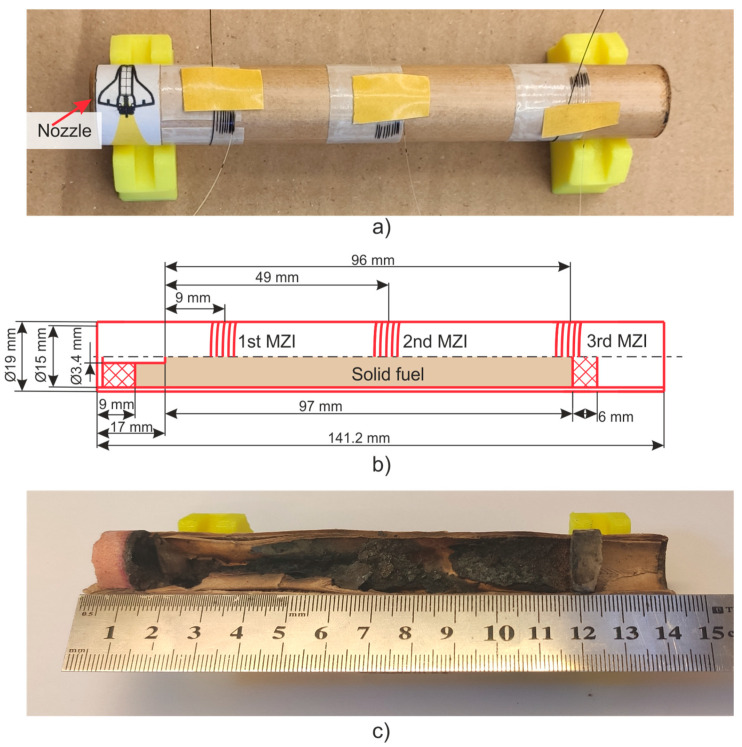
View of the model SRM E-5-0 with installed MZI (**a**), a diagram with dimensions between the main SRM components and the MZI (**b**), and the SRM section after work (**c**).

**Figure 4:**
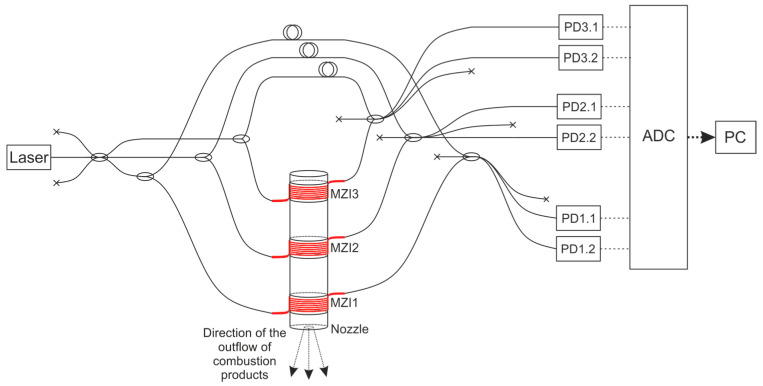
Scheme of the experimental setup. Laser: radiation source; PD: photodiode; ADC: analog-digital converter (L-Card Е20-10); PC: personal computer.

In this study, a model SRM E-5-0 is the research object. It operates via a black powder [53], that was pressed into a cylindrical body made of cardboard. A graphite nozzle block was installed on the bottom with a critical section diameter of 3.4 mm. Fuel ignition was performed using a 0.5 g black powder sample via a combustible wire. The model SRM characteristics are shown in Table 1. Figure 3 shows the SRM photograph (a), a diagram with dimensions between the main components and the MZIs (b), and a section of the SRM after the study was conducted (c). The experimental setup included three MZIs; its scheme is shown in Figure 4. The MZI sensing arms were fixed equidistantly along the entire fuel length.

**Figure 5:**
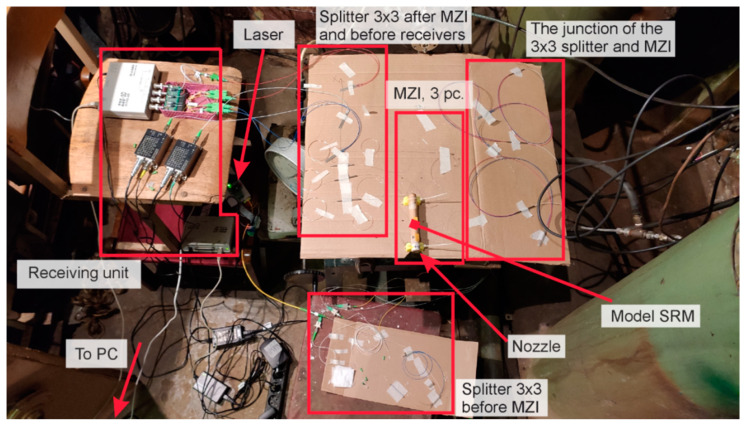
Photo of the assembled laboratory setup.

A narrow-band NKT BASIK MIKRO fiber laser with a central wavelength of 1550 nm and a bandwidth of less than 0.1 kHz was used. Its radiation emitted through a 3 × 3 splitter to three independent equal-arm MZIs. The supporting arms were at rest, and the sensing arms were coiled on the SRM housing (see Figure 1). The measuring arm of each MZI consisted of *L*_sens_ = *Nπd =* 10·*π*·19 mm ≈ 0.6 m of SMF-28. We glued this fiber loop-to-loop using one layer of double-sided tape. This method of construction increased the MZI sensitivity to fluctuations of housing diameter [54]. The SRM was fixed on the metal table by clamps. Such mounting proved adequate to complete the measurements; the motor shifted slightly at the start as a result of the highest pressure, and the sensing fiber remained connected to the housing at all times. The process was recorded, and is provided in the attached Video S1. Two fibers of each MZI 3 × 3 splitter outputs were transferred to photodiodes (PD). The signals were digitized on an ADC with a sampling rate of 2.5 MHz. This value determined the maximum detectable vibration frequency (1.25 MHz), according to the Nyquist theorem. An image of a laboratory setup with the measurement and registration systems is shown in Figure 5. Before the experiment, we checked the setup’s integrity and its ability of deformation registration.

### 3.2. Analysis of the Investigation Results

**Figure 6:**
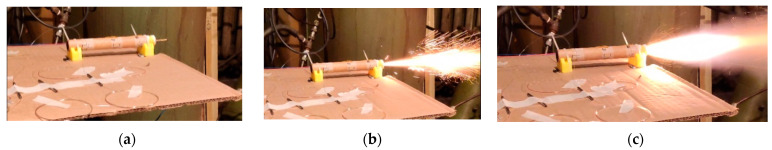

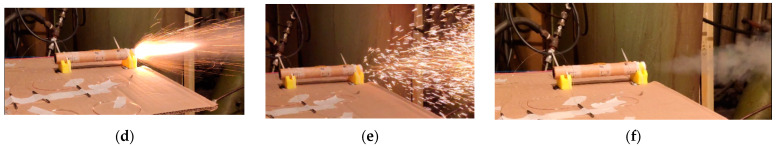
Photos of the experiment: before the beginning (**a**); launch (**b**); nominal mode (**c**,**d**); engine shutdown and combustion termination (**e**,**f**).

Images of the SRM stages are shown in Figure 6, including start-up (a,b), operation in nominal mode (c,d), and shutdown (e,f). It is worth noting that the tracks of the condensed phase particles flowed out of the engine nozzle, which is common for the combustion products of powder and metal-containing fuels. The total operating time was about 5.5 s, during which the optical fiber did not undergo any damage or changes due to the effect of high-temperature combustion products.

**Figure 7:**
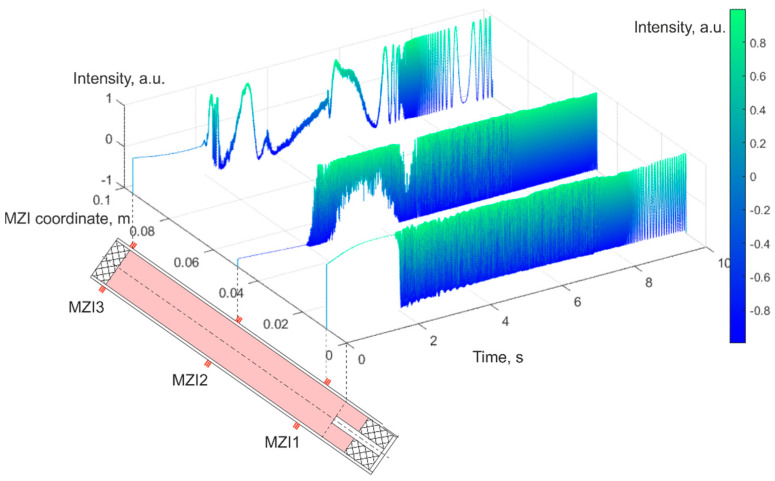
Plots of one data channel for each MZI.

The recorded data from each MZI were processed in the time and frequency domains. The data ranges from 2 seconds before fuel ignition through the combustion process to around 2 seconds after its completion. Until the engine was turned on, the signal at each PD changed with a small amplitude. The high-frequency component occurred due to the PD and the laser phase noise, and the low-frequency fluctuation was a result of the installation temperature drift and the laser wavelength drift. At launch, the amplitude increased in signal oscillations on all PDs. An example of the initial data from one channel of each MZI is presented in Figure 7—the oscillation amplitude increased on all interferometers from the moment the engine was launched, but it only reached the maximum contrast when the combustion surface of the solid fuel reached the MZI sensing arm on the SRM housing.

**Figure 8:**
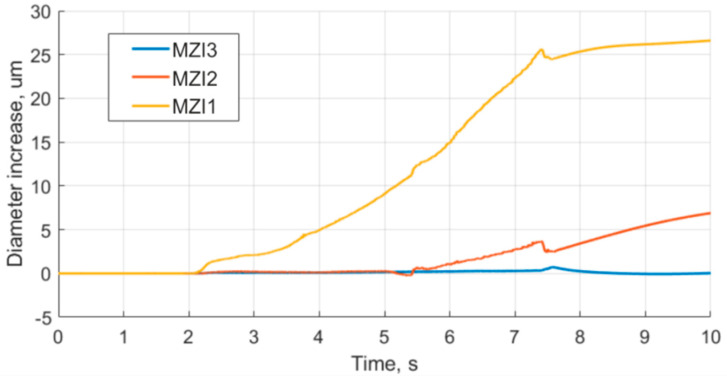
Plots of housing diameter increase.

For each sensor, the phase-unwrapping procedure was carried out according to Formula (2). An absolute value of the optical signal phase change, from the initial state (before the engine launch), was obtained and was found to be proportional to the change of the fiber length on the model SRM according to Equation (3). The results highlight that the closer the MZI to the nozzle, the more changes it experienced. The plots for the housing diameter increase are presented in Figure 8.

**Figure 9:**
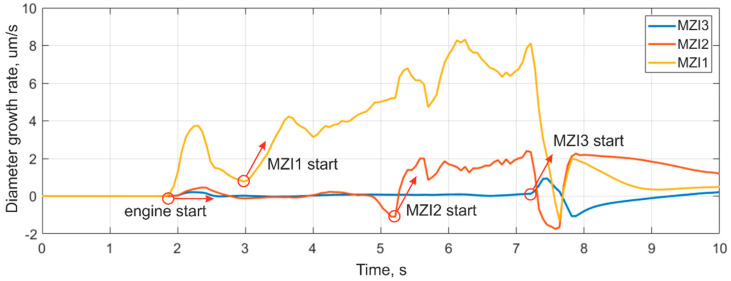
Derivative plots of increasing diameter.

The derivatives of the housing diameter expansion graphs were calculated with a 60 ms window, allowing for the exclusion of high-frequency oscillations and their influence on the derivative stability. All of the sensors had a moment of initial expansion at the engine start, after which the diameter value became relatively stable, without a noticeable trend of expansion. Graphs illustrating the derivatives from each sensor are shown in Figure 9. A sharp increase in the derivative was observed when the combustion surface coordinate reached the sensor fixing point. These points are marked with circles in Figure 9.

**Figure 10:**
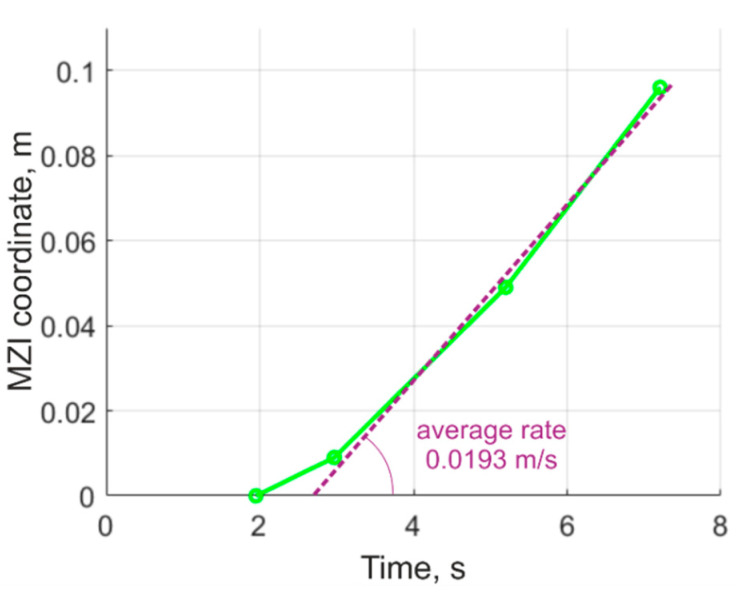
Graph of the combustion surface movement.

The coordinates of the combustion-front propagation were determined by the time of derivative sharp growth for the sensors, and are shown in Table 2. Based on these values, we graphed the combustion surface movement, as presented in Figure 10.

The burning rate is non-linear in the first and smaller section of the graph due to the uneven combustion front, caused by the presence of a groove at the end of the solid fuel, as well as the combustion of the igniter sample, also made of black powder. After the end of the ignition period, the time dependence of the combustion surface movement was found to be close to linear with an average linear displacement velocity of 0.0193 m/s.

**Figure 11:**
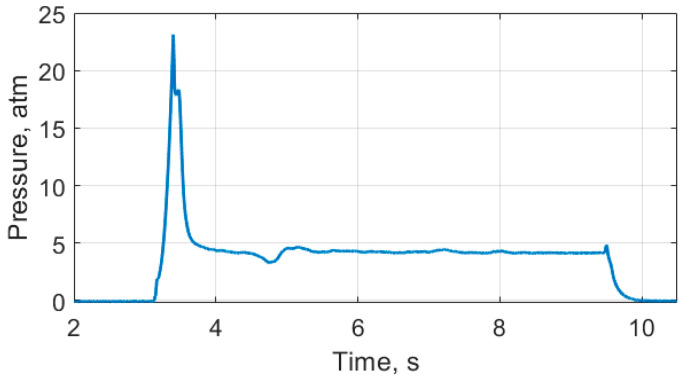
Pressure plot for the similar SRM.

This dependence (nonlinear during the ~1 second and then linear) is consistent with the results of a similar SRM test, showing that the pressure in the combustion chamber after around 1 second, following the engine start, became almost constant, as shown in Figure 11. The pressure in the combustion chamber during the experiment was measured using a special setup. The SRM was installed in a stainless steel external chamber with a pressure sensor. This setup was the only method by which to fix the pressure sensor to SRM. The described modification slightly increased the combustion time to 6.5 s. However, in general, the pressure change during the investigation remained unchanged for all SRMs of such a model. The fuel and housing construction provide a constant combustion surface area for when the fuel burns, therefore, a constant pressure in the chamber after ~1 second after start becomes apparent even in the presence of deviations in the initial temperature, solid fuel composition, critical section diameter, etc.

**Figure 12:**
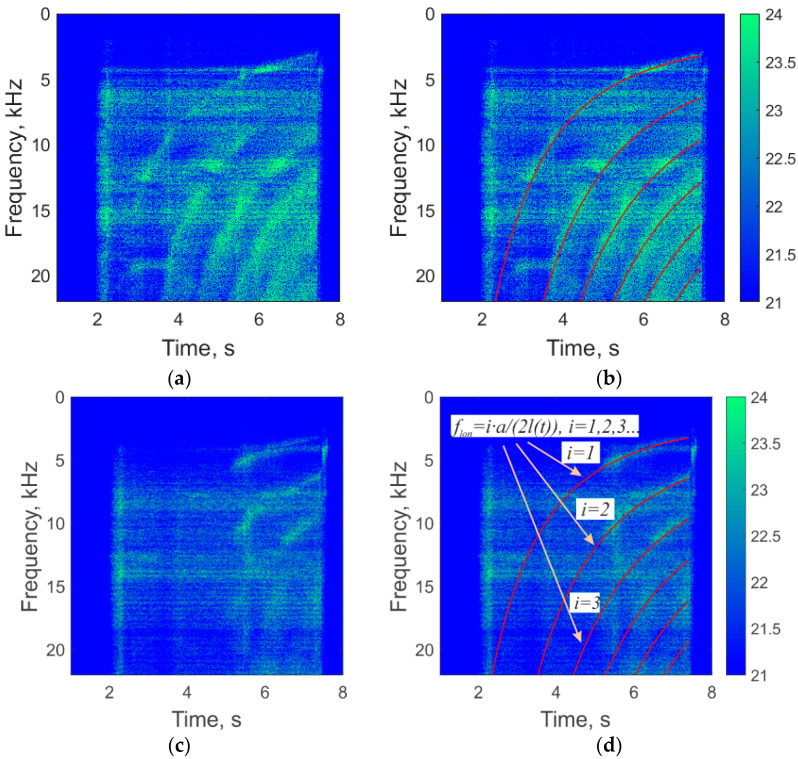

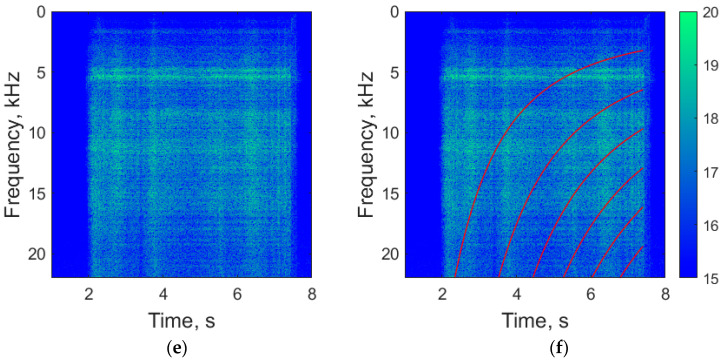
Spectrograms of the housing diameter deformation signals recorded by sensors: pure graphs MZI1 (**a**), MZI2 (**c**), MZI3 (**e**), and the same with marked ideal longitudinal mode and its harmonics for MZI1 (**b**), MZI2 (**d**), and MZI3 (**f**).

Spectrograms of the unwrapped signal were calculated for each sensor to complete the frequency analysis. They are shown in Figure 12. Some peaks in the characteristic frequencies can be expected. The values of the first longitudinal *f_lon_*, tangential *f_tan_*_,_ and radial *f_rad_* modes of chamber sound vibrations, according to Equation (4), are as follows:

$$[\begin{array}{c} \begin{array}{c} f_{lon}=\text{from}\;45\;\text{kHz}\;\text{at}\;\text{SRM}\;\text{start}\;\text{to}\;3.30\;\text{kHz}\;\text{at}\;\text{finish}\\ f_{tan}=28.13\;\text{kHz} \end{array}\\ f_{rad}=58.56\;\text{kHz} \end{array}$$

A shifting peak, in the range from 3 to 20 kHz, and its harmonics are visible in the spectrograms, and have been caused by the changing longitudinal modes. They have a lower frequency in comparison to tangential and radial modes, so longitudinal modes were the most probable. It is possible to calculate the speed of sound, which is determined by the used fuel. Based on the boundary conditions—the minimum frequency of the longitudinal mode in Figure 12a is 3.3 kHz, and the length of the combustion chamber, which was 109 mm—the following results are found using Equation (4):

$$a=2l\left(t\right)f_{lon}\left(t\right)=2l\left(7.4\right)f_{lon}\left(7.4\right)=2\cdot 0.109\cdot 3300\approx 720m/s$$

**Figure 13:**
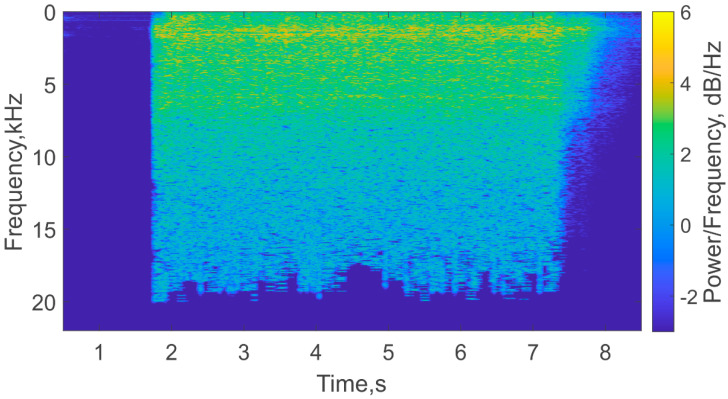
Spectrogram of the audio signal recorded during the experiment.

The spectrograms in Figure 12 show that the peak of longitudinal oscillations only appeared in the interferometer signal when the burning front reached the MZI fixing point. We also analyzed the spectrogram of the audio signal, which was recorded by a microphone during the experiment, and is presented in Figure 13. This plot did not reveal any changing peaks during the burning process. This highlights the advantage provided by the FOS, which was able to detect vibrations that have been generated via sound longitudinal modes. Thus, the fiber MZI worked as a small, light, fire-safe, and easily installed sensor for SRM monitoring.

We plotted the graphs of the ideal longitudinal vibration modes with a known speed of sound and the length of the combustion chamber, calculated according to Equation (4). They are shown in Figure 12b,d,f and are in good agreement with the experimental data. The length changes of the combustion chamber account for two aspects. The first is a meniscus of the burning front, among other factors, caused by a deepening in the solid fuel, as shown in Figure 2. The second is a partial burnout of the plug with the nozzle, from 9 to 5 mm in the center, as shown in Figure 2b. It should be noted that the frequencies of the peaks on the MZI1 and MZI2 spectrograms coincide during their occurrence.

The graphs obtained allow us to conclude that the rate of fuel burnout in the model SRM was almost constant, since the burning front reached the coordinate of the MZI1 after 1 s following initiation.

## 4. Discussion

A non-invasive diagnostic technique using fiber-optic MZIs as sensors has been developed. This technique makes it possible to determine the characteristics of intra-chamber processes—particularly the burning rate of solid fuel and the length of the combustion chamber—at a given time. The calculations aim to determine when the burning front passes through the MZI fixation points. For each MZI, this can be determined by the derivative growth. Additionally, the resonance frequencies of the acoustic vibration longitudinal modes in the combustion chamber can be determined via the shifting peaks in the spectrogram. As a result, the calculation of the combustion chamber length and the burning rate of solid fuel during the overall SRM worktime can be performed.

For the tested SRM, an uneven combustion of the fuel was detected during the first phase of the work due to deepening occurring at the point at which combustion begins. Then, the burnout rate became almost constant; for our experimental conditions, the burnout rate was approximately 0.0193 m/s.

## Figures and Tables

**Table 1:** Characteristics of the model SRM E-5-0 used in the work.

Parameter	Value
External diameter, mm	19.0
Internal diameter, mm	15.0
Full length, mm	141.2
Burning time of a solid fuel charge, s	5.5 ± 0.1
Solid fuel charge length, mm	105
Fuel	black powder (C_11.362_H_6.493_O_22.768_N_7.319_S_3.243_K_7.319_)

**Table 2:** Time and coordinates of burning front propagation.

Point	Fuel Bottom Edge	MZI1	MZI2	MZI3
Coordinate, mm	0	9	49	96
Time of the derivative growth beginning	1.95	2.97	5.20	7.21

## Data Availability

The data presented in this study are available on request from the corresponding author.

## Supplementary Materials

The following are available online at https://www.mdpi.com/article/10.3390/s21237836/s1, Video S1: Process of SRM work.
